# Adiponectin, IGFBP-1 and -2 are independent predictors in forecasting prediabetes and type 2 diabetes

**DOI:** 10.3389/fendo.2022.1092307

**Published:** 2023-01-05

**Authors:** Kerstin Brismar, Agneta Hilding, Ishrath Ansurudeen, Allan Flyvbjerg, Jan Frystyk, Claes-Göran Östenson

**Affiliations:** ^1^ Department of Molecular Medicine and Surgery, Rolf Luft Research Center for Diabetes and Endocrinology, Karolinska Institutet, Stockholm, Sweden; ^2^ Department of Endocrinology, Diabetes and Metabolism, Karolinska University Hospital, Stockholm, Sweden; ^3^ Steno Diabetes Center Copenhagen (SDCC), the Capital Region of Denmark and Department of Clinical Medicine, Faculty of Health and Medical Sciences, University of Copenhagen, Copenhagen, Denmark; ^4^ Department of Clinical Medicine, Health, Aarhus University, Aarhus C, Denmark; ^5^ Endocrine Research Unit, Department of Endocrinology, Odense University Hospital & Department of Clinical Medicine, Faculty of Health Sciences, University of Southern Denmark, Odense, Denmark

**Keywords:** biomarkers, prospective study, abnormal glucose tolerance, adiponectin, IGFBP-1, IGFBP-2, gender

## Abstract

**Objective:**

Adiponectin and insulin-like growth factor (IGF) binding proteins IGFBP-1 and IGFBP-2 are biomarkers of insulin sensitivity. IGFBP-1 reflects insulin sensitivity in the liver, adiponectin in adipose tissue and IGFBP-2 in both tissues. Here, we study the power of the biomarkers adiponectin, IGFBP-1, IGFBP-2, and also included IGF-I and IGF-II, in predicting prediabetes and type 2 diabetes (T2D) in men and women with normal oral glucose tolerance (NGT).

**Design:**

Subjects with NGT (35-56 years) recruited during 1992-1998 were re-investigated 8-10 years later. In a nested case control study, subjects progressing to prediabetes (133 women, 164 men) or to T2D (55 women, 98 men) were compared with age and sex matched NGT controls (200 women and 277 men).

**Methods:**

The evaluation included questionnaires, health status, anthropometry, biochemistry and oral glucose tolerance test.

**Results:**

After adjustment, the lowest quartile of adiponectin, IGFBP-1 and IGFBP-2 associated independently with future abnormal glucose tolerance (AGT) in both genders in multivariate analyses. High IGFs predicted weakly AGT in women. In women, low IGFBP-2 was the strongest predictor for prediabetes (OR:7.5), and low adiponectin for T2D (OR:29.4). In men, low IGFBP-1 was the strongest predictor for both prediabetes (OR:13.4) and T2D (OR:14.9). When adiponectin, IGFBP-1 and IGFBP-2 were combined, the ROC-AUC reached 0.87 for women and 0.79 for men, higher than for BMI alone.

**Conclusion:**

Differences were observed comparing adipocyte- and hepatocyte-derived biomarkers in forecasting AGT in NGT subjects. In women the strongest predictor for T2D was adiponectin and in men IGFBP-1, and for prediabetes IGFBP-2 in women and IGFBP-1 in men.

## Introduction

Type 2 diabetes (T2D) is a multi-factorial disease represented by insulin resistance, impaired insulin secretion and hyperglycemia with obesity as the most common cause of insulin resistance. Although insulin production and sensitivity are the primary regulators of glucose homeostasis, other glucose-regulating factors are likely to play a role in the development of abnormal glucose tolerance (AGT). In this context, components of the insulin-like growth factor (IGF) system, which exert glucose metabolic effects, have been in focus (1–11). Recent studies showed that subjects with either low or high serum IGF-I levels are at increased risk of developing T2D (5, 7, 8). IGF-II has also been suggested to be involved in metabolic disorders and diabetes (4, 9). The IGF binding proteins, IGFBP-1 and -2, may influence glucose regulation and homeostasis by modulating IGF bioavailability (1, 2, 10, 11). Circulating IGFBP-1, mainly produced in the liver where insulin suppresses its production, reflects insulin production, hepatic and whole-body insulin sensitivity (2, 11–14). Low IGFBP-1 concentrations are predictive of future development of T2D (5, 15–18) and a stronger predictor of T2D than fasting plasma insulin and glucose (16, 17). IGFBP-2 is secreted by both liver and adipose tissues, and its secretion is regulated by leptin and nutrition (19–21). Serum IGFBP-2 is associated with insulin sensitivity with levels inversely correlated with plasma insulin and free IGF-I (18–20). Furthermore, low serum IGFBP-2 associates with increased fat mass, elevated triglyceride levels and increased risk of T2D (6, 18, 19, 21–23).

Adiponectin is an insulin-sensitizing peptide, secreted mainly by adipocytes (24). Serum levels of adiponectin are reduced in obese subjects with increased visceral fat (25). Adiponectin is primarily regulated by inflammatory cytokines and angiogenic factors. There is a close association between low adiponectin levels and future as well as present T2D (24–28).

In a recent study, a panel of six biomarkers including inflammatory markers, HDL cholesterol, IGFBP-2 and adiponectin improved prediction of future T2D when added to a clinical model including HbA1c, which was used instead of fasting glucose and insulin (29). However, according to baseline HbA1c levels the studied cohort included subjects with prediabetes. In the study by Schiffman et al., an insulin resistances score (IRScore) based on insulin and C-peptide improved diabetes risk assessment in older European subjects compared to established risk factors plus fasting glucose at baseline. The IRScore predicted T2D regardless of prediabetes (30).

The aim of our prospective study of middle-aged NGT healthy subjects, many with a family history of diabetes (FHD), was to investigate the predictive power of the liver and adipose tissue specific biomarkers of insulin sensitivity, IGFBP-1, IGFBP-2 and adiponectin, as well as of IGF-I and IGF-II, to forecast development of prediabetes and T2D. Moreover, we thought it was of interest to study men and women separately, since many of these biomarkers express gender differences.

## Material and methods

### Study population

This is a nested case control study based on a larger prospective cohort. The study population participated in an epidemiological survey, the Stockholm Diabetes Prevention Program. The design of the study has been described previously (31). Briefly, individuals aged 35-56 years without known diabetes were recruited for the baseline study performed in 1992-94 for men and in 1996-98 for women. Due to the study design, a selection was made so that the study cohort was enriched with participants with FHD (see **Supplemental Flow Diagram**). Thus, at baseline about 50% of the participants had a positive FHD, defined as known diabetes in at least one first-degree relative (father, mother, brother or sister) or at least two second-degree relatives (grand-parents, uncles or aunts). Eight to ten years later; in 2002-2004 for men and in 2004-2006 for women, a follow-up study was undertaken. All baseline participants were invited with the exception of those, who were diagnosed with T2D at baseline, had moved out of the Stockholm area or were deceased. In total 4,821 women and 3,128 men participated in the baseline study. Corresponding figures for the follow-up study were 3,329 (76% of invited) and 2,383 (87% of invited), respectively. Baseline and follow-up studies consisted of an extensive questionnaire covering lifestyle factors, a health examination including measurements of blood pressure, weight, height, hip and waist. Blood samples were taken after an overnight fast and 2-h after drinking 75 g glucose (oral glucose tolerance test (OGTT)). Individuals, who progressed from normal glucose tolerance (NGT) at baseline to AGT at follow-up, were selected as cases. Thus, cases consisted of persons who developed prediabetes (IGT and IFG+IGT) (133 women, 164 men) or overt T2D (55 women, 98 men). A similar number of controls were randomly selected among subjects having NGT and negative FHD at both baseline and follow-up (200 women, 277 men), matched to cases by sex and age in five-year interval. Only subjects having data on all variables were included in the analyses.

The study, approved by the Ethics Committee at Karolinska University Hospital, was carried out in accordance with the Declaration of Helsinki. All participants gave informed consent.

### Classification of glucose tolerance

Individuals were categorized after an OGTT according to 1999 WHO criteria (WHO 1999) as having NGT, impaired fasting glucose (IFG, between 6.1 and 7.0 mmol/L), impaired glucose tolerance (IGT, 2-h value between 7.8 and 11.1 mmol/L) or T2D.

Subjects having IGT or IFG plus IGT were defined as having prediabetes. Moreover, subjects classified as having T2D at follow-up are those diagnosed at the follow-up examination as well as those, who were diagnosed by a physician during the period between baseline and follow-up examinations. *The physicians in primary care diagnosed T2D when the fasting glucose levels were higher than 7 mmol/l at two or more occasions or symptoms accompanied by glucose levels higher than 11.1 mmol/l or HbA1c above 47 mmol/mol. All subjects categorized as having prediabetes in the present study were newly diagnosed by the OGTT at the follow up occasion.*

### Classification of established diabetes risk factors and potential confounders

Body mass index (BMI, kg/m^2^) was divided into three groups (<25.0, 25.0-29.9 and ≥30.0). Similarly, waist was categorized in three groups: <80, 80-87, and >87 cm for women, and <94, 94-101, and >101 cm for men. Physical activity during leisure time and smoking, based on the response alternatives were categorized into three groups (**Table 1**). Socioeconomic position, based on self-reported occupation, classified according to the standard system from Statistics Sweden, was categorized into four groups (unskilled/skilled manual workers, low-level non-manual employees, medium- and high-level non-manual employees and self-employed/farmers). Education comprised three categories; low (elementary school), middle (senior high school, technical and vocational school), and high (college, university). Blood pressure was dichotomized into normal (diastolic and systolic blood pressure below 90 and 140 mmHg, respectively, without hypertension treatment) and high (systolic blood pressure ≥140 and/or diastolic blood pressure ≥ 90 mmHg and/or on anti-hypertensive treatment).

**Table 1:** Characteristics at baseline in women and men according to glucose tolerance at follow-up.

	Controls mean (95% CI)	Future Prediabetes mean (95% CI)	Future T2D mean (95% CI)	p-value^a^
**WOMEN**, n	200	133	55	
Age (yrs)	49.0 (48.4-49.6)	49.1 (48.4-49.8)	49.3 (48.1-50.5)	0.899
BMI (kg/m^2^)	24.4 (23.8-25.0)	27.6 (26.8-28.4)***	29.9 (28.3-31.6)***++	<0.001
Waist (cm)	78 (77-79)	86 (85-88)***	92 (89-96)***++	<0.001
Systolic BP (mm Hg)	119 (117-121)	128 (125-131)***	128 (124-131)**	<0.001
Diastolic BP (mm Hg)	74 (72-75)	80 (78-81)***	79 (77-81)**	<0.001
Glucose, fasting (mmol/l)	4.6 (4.5-4.6)	4.9 (4.9-5.0)***	5.2 (5.1-5.3)***++	<0.001
Glucose, 2h (mmol/l)	4.3 (4.2-4.4)	5.7 (5.5-5.9)***	5.8 (5.4-6.1)***	<0.001
Insulin, fasting (pmol/l)^b^	54 (51-57)	73 (68-79)***	91 (81-103)***++	<0.001
Insulin, 2h (pmol/l)^b^	173 (162-185)	289 (261-319)***	352 (297-419)***	<0.001
IGFBP-1, fasting (µg/l)^b^	43 (40-46)	29 (26-32)***	24 (21-28)***	<0.001
IGFBP-1, 2h (µg/l)^b^	22 (20-23)	15 (14-16)***	14 (12-15)***	<0.001
IGFBP-2 (µg/l)^b^	234 (221-248)	153 (141-165)***	144 (127-162)***	<0.001
Adiponectin (mg/l)^b^	13.9 (13.2-14.7)	10.0 (9.3-10.7)***	8.6 (7.8-9.5)***	<0.001
IGF-I (µ/l)^b^	175 (169-182)	186 (178-195)	168 (155-183)	0.038
IGF-II (µ/l)^b^	799 (778-820)	834 (807-862)	796 (755-839)	0.106
Physical activity, n (%)				
sedentary	19 (9.5)	23 (17.3)	16 (29.1)	
moderate	109 (54.5)	85 (63.9)	28 (50.9)	
regular	72 (36.0)	25 (18.8)	11 (20.0)	<0.001
Smoking, n (%)				
never	82 (41.0)	57 (42.8)	16 (29.1)	
former	67 (33.5)	38 (28.6)	22 (40.0)	
current	51 (25.5)	38 (28.6)	17 (30.9)	0.384
Socioeconomic status, n (%)				
low	45 (22.5)	42 (31.6)	17 (30.9)	
middle	44 (22.0)	38 (28.6)	21 (38.2)	
high	106 (53.0)	50 (37.6)	14 (25.4)	
self-employed	5 (2.5)	3 (2.2)	3 (5.4)	0.006
Hypertension^c^, n (%)				
no	167 (83,5)	89 (66.9)	38 (69.1)	
yes	33 (16.5)	44 (33.19	17 (30.9)	0.001
**MEN**, n	277	164	98	
Age (yrs)	47.6 (47.0-48.2)	47.6 (46.8-48.3)	47.8 (46.9-48.8)	0.932
BMI (kg/m^2^)	25.2 (24.8-25.5)	27.5 (27.0-28.1)***	27.6 (26.9-28.4)***	<0.001
Waist (cm)	91 (90-92)	96 (94-97)***	96 (94-98)***	<0.001
Systolic BP (mm Hg)	123 (121-125)	131 (128-133)***	130 (127-133)***	<0.001
Diastolic BP (mm Hg)	79 (78-80)	83 (82-85)***	82 (80-84)**	<0.001
Glucose, fasting (mmol/l)	4.5 (4.5-4.6)	4.8 (4.6-4.8)***	5.0 (4.9-5.1)***++	<0.001
Glucose, 2h (mmol/l)	4.4 (4.2-4.5)	5.2 (5.1-5.4)***	5.6 (5.4-5.9)***+	<0.001
Insulin, fasting (pmol/l)^b^	99 (94-103)	121 (113-129)***	131 (121-143)***	<0.001
Insulin, 2h (pmol/l)^b^	247 (231-264)	411 (374-451)***	397 (346-455)***	<0.001
IGFBP-1, fasting (µg/l)^b^	29 (27-31)	14 (13-16)***	14 (13-16)***	<0.001
IGFBP-1, 2h (µg/l)^b^	15 (14-16)	6 (6-7)***	7 (6-8)***	<0.001
IGFBP-2 (µg/l)^b^	185 (176-195)	131 (121-142)***	130 (117-144)***	<0.001
Adiponectin (mg/l)^b^	8.6 (8.2-9.0)	7.5 (7.0-8.0)**	7.0 (6.4-7.6)***	<0.001
IGF-I (µ/l)^b^	185 (179-191)	187 (179-195)	185 (175-196)	0.919
IGF-II (µ/l)^b^	939 (918-961)	959 (929-989)	995 (956-1037)*	0.043
Physical activity, n (%)				
sedentary	21 (7.6)	15 (9.1)	14 (14.3)	
moderate	125 (45.1)	101 (61.6)	54 (55.1)	
regular	131 (47.3)	48 (29.3)	30 (30.6)	<0.001
Smoking, n (%)				
never	117 (42.2)	57 (34.8)	30 (30.6)	
former	104 (37.5)	64 (39.0)	29 (29.6)	
current	56 (20.2)	43 (26.2)	39 (39.8)	0003
Socioeconomic status, n (%)				
low	82 (29.6)	54 (32.9)	33 (33.7)	
middle	42 (15.2)	33 (20.1)	23 (23.5)	
high	133 (48.0)	71 (43.3)	37 (37.8)	
self-employed	20 (7.2)	6 (3.7)	5 (5.1)	0.240
Hypertension^c^, n (%)				
no	209 (75.5)	93 (56.7)	53 (54.1)	
yes	68 (24.5)	71 (43.3)	45 (45.9)	<0.001

^a^Comparison between groups was for continuous variables performed by ANOVA, and if significant followed by Scheffé post-hoc test: *P<0.05, **P<0.01,

***P<0.001 vs control; +P<0.05, ++P<0.01, +++P<0.001 vs prediabetes, and for categorical variables by chi-square test. ^b^geometric mean ^c^hypertension; no=normal blood pressure and no hypertension treatment, yes=high blood pressure and/or hypertension treatment. All subjects had normal glucose tolerance (NGT) at baseline. The Controls stayed NGT at follow up.

### Assays

All samples were assayed in duplicate. Serum IGFBP-1 was measured by an in-house radioimmunoassay (RIA) using a polyclonal antibody and human IGFBP-1 as standard (32). Intra- and inter-assay CV were 3% and 10%, respectively. Serum IGFBP-2 was determined by an in-house time-resolved immunofluorometric assay (TR-IFMA) based on commercial reagents as previously described, with intra- and inter-assay CVs averaging 5 and 12%, respectively (33). Serum adiponectin was determined by an in-house TR-IFMA based on commercial antibodies as previously described, with intra- and inter-assay CVs averaging <5 and <13%, respectively (34). This assay was later demonstrated to detect all major forms of adiponectin in human serum (high-molecular, medium molecular and low molecular weight forms) (34). Serum IGF-II was determined following acid ethanol extraction of serum, using a highly sensitive TR-IFMA as previously described, intra- and inter-assay CVs averaging <5 and <15%, respectively (35). IGF-I was measured in serum by RIA after acid-ethanol extraction and cryo-precipitation, using des-(1-3)-IGF-I as tracer to minimize interference by IGFBPs (36). Intra- and inter-assay CV were 4% and 11%, respectively.

Venous serum glucose was assayed using the glucose oxidase method (Yellow Springs Glucose Analyzer, Yellow Springs, OH, USA).

Immunoreactive insulin was assayed by in-house RIA, using a polyclonal antibody, human insulin as standard and charcoal addition to separate antibody-bound and free insulin. The intra-assay CV was 5.8-8.4% and the inter-assay CV was 11.5-16.9%. In this assay proinsulin has a 100% cross reactivity (37).

### Data analysis

Results are presented separately for women and men. Clinical characteristics are presented as means and 95% confidence intervals (CI). Variables not normally distributed (i.e. adiponectin, IGFBP-1, IGFBP-2, IGF-I, IGF-II and insulin) were log-transformed prior to analyses. Differences between more than two groups were for continuous variables analyzed by one-way ANOVA and if significant, followed by Scheffé *post hoc* test and for categorical variables by chi-square test. Paired t-test was performed when comparing two occasions within group and unpaired t-test when comparing two groups. Odds ratios (ORs) together with 95% confidence intervals (CIs) were calculated by multiple logistic regression analyses to explore associations between serum variables and an abnormal glucose regulation.

Potential confounders (BMI, waist, physical activity during leisure time, smoking, socioeconomic position, education and blood pressure) were tested separately in logistic regression models including the exposure variable and age. The change-in-estimate method was used, meaning that variables that contributed to at least a 10% change of the age-adjusted crude estimate in any of the outcome measures were included in the final multi-adjusted model. Waist and BMI, just like socioeconomic position and education, had similar influence on the crude estimates and accordingly BMI and socioeconomic position were chosen for the final model. Thus, two logistic regression models are given; model 1 adjusted for age and model 2 adjusted for age, BMI, physical activity, smoking, socioeconomic position and blood pressure.

In the regression model serum variables are categorized in quartiles according to the distribution within sex or used as continuous ^2^log-transformed variables and thus reported per halving (adiponectin, IGFBP-1 and IGFBP-2), or per doubling (IGF-I and IGF-II). Co-linearity between biomarkers was evaluated by estimating variance inflation factor (VIF) which was found to not exceed 2.0.

Receiver operating characteristics (ROC) curve analyses were used to evaluate and compare the predictive power of the biomarkers. In PROC LOGISTIC (SAS statistical package) the ROCCONTRAST statement, implementing a nonparametric approach, was used to analyze differences between the respective areas under the curve (AUC). The analyses were performed using SAS statistical package version 9.2 for Windows (SAS Institute Inc., Cary, NC, USA) and Statistica StatSoft version 10 (Tulsa, OK, USA). P-values <0.05 were considered statistically significant.

## Results

### Women

At baseline, all subjects had normal fasting glucose, OGTT and blood pressure. Significant differences were observed in all continuous parameters except for age and serum IGF-II levels in those who later developed prediabetes or T2D compared to the controls, who remained NGT (**Table 1**). FHD was reported at baseline in 71% of women, who later developed prediabetes, and in 80% of women, who developed T2D. BMI and waist circumference were at baseline progressively higher in those, who later developed prediabetes or T2D. Conversely, baseline levels of adiponectin, IGFBP-1 and IGFBP-2 were significantly lower in individuals, who later developed prediabetes or T2D compared to the controls.

Those who later developed T2D were at baseline more sedentary with less regular physical activity and they had lower socioeconomic status compared to the controls (**Table 1**). Hypertension was more common in those who later developed prediabetes or T2D.

Eight years later at follow up the number with known FHD had increased to 75% of those with prediabetes and to 84% of those with T2D.

After adjustment for known risk factors, significant ORs for future T2D were obtained for the lowest quartiles of adiponectin (OR:29.42), IGFBP-2 (OR:9.52) and IGFBP-1 (OR:5.41), and for the highest quartile of IGF-I (OR:3.79). IGF-II did not predict T2D. For the prediction of prediabetes, significant ORs were obtained for all five proteins, the strongest for IGFBP-2 (OR:7,48) and adiponectin (OR:6,06) **(** **Table 2**).

**Table 2:** Odds ratios (ORs) for decreasing baseline values of IGFBP-2, adiponectin and IGFBP-1 and increasing baseline values of IGF-I and IGF-II in subjects having normal glucose tolerance (NGT) at baseline in the association to development of prediabetes and type 2 diabetes at follow-up compared to remaining NGT.

WOMEN								MEN							
	NGT	Prediabetes			Type 2 diabetes				NGT	Prediabetes			Type 2 diabetes		
	n	n	OR	95% CI	n	OR	95% CI		n	n	OR	95% CI	n	OR	95% CI
**IGFBP-2,** μg/l								**IGFBP-2,** μg/l							
>268	79	14	1.00		5	1.00		>224	92	28	1.00		14	1.00	
193-268	52	34	2.70	1.25-5.84	8	0.92	0.22-3.87	161-224	95	27	0.96	0.50-1.83	17	1.37	0.59-3.17
141-192	47	32	2.15	0.97-4.79	16	2.81	0.77-10.26	110-160	58	48	2.54	1.34-4.82	29	4.39	1.89-10.23
<141	22	53	7.48	3.17-17.64	26	9.52	2.59-35.03	<110	32	61	4.03	1.97-8.25	38	7.63	3.13-18.59
continuous, ^2^log	200	133	3.74	2.32-6.03	55	5.31	2.43-11.58	continuous, ^2^log	277	164	2.46	1.68-3.60	98	3.17	1.99-5.07
**Adiponectin**, mg/l								**Adiponectin**, mg/l							
>15.62	74	18	1.00		5	1.00		>10.55	84	36	1.00		17	1.00	
11.60-15.62	62	28	1.48	0.69-3.16	5	1.19	0.25-5.69	7.95 - 10.55	70	41	1.17	0.64-2.14	20	1.33	0.60-2.91
8.56-11.59	43	42	2.63	1.24-5.57	14	5.98	1.42-25.16	6.04 – 7.94	74	36	0.96	0.52-1.75	27	1.56	0.73-3.32
<8.56	21	45	6.06	2.71-13.57	31	29.42	7.11-121.77	<6.04	49	51	1.70	0.93-3.12	34	2.35	1.11-4.99
continuous, ^2^log	200	133	3.30	2.04-5.33	55	10.89	4.41-26.91	continuous, ^2^log	277	164	1.46	1.02-2.10	98	1.92	1.23-2.98
**IGFBP-1,** μg/l								**IGFBP-1,** μg/l							
>49	73	20	1.00		5	1.00		>35	106	18	1.00		9	1.00	
35-49	63	28	1.49	0.71-3.10	8	1.60	0.42-6.04	23 - 35	88	23	1.62	0.79-3.31	20	3.00	1.22-7.35
24-34	42	39	2.83	1.35-5.96	18	4.84	1.36-17.19	14 - 22	57	47	4.16	2.10-8.21	29	6.33	2.56-15.62
<24	22	46	4.27	1.86-9.79	24	5.41	1.45-20.12	<14	26	76	13.44	6.33-28.54	40	14.89	5.61-39.48
continuous, ^2^log	200	133	2.17	1.47-3.20	55	2.98	1.62-5.48	continuous, ^2^log	277	164	3.23	2.39-4.35	98	3.19	2.20-4.60
**IGF-I,** μg/l								**IGF-I,** μg/l							
<150	52	28	1.00		18	1.00		<161	63	42	1.00		32	1.00	
150-182	57	29	0.94	0.44-2.01	12	0.62	0.21-1.81	161 - 185	82	36	0.60	0.32-1.10	17	0.32	0.15-0.68
183-214	53	32	1.42	0.67-2.98	12	1.14	0.38-3.38	186 - 218	62	42	1.10	0.60-2.04	22	0.71	0.33-1.53
>214	38	44	3.79	1.76-8.15	13	3.37	1.04-10.95	>218	70	44	0.81	0.43-1.53	27	0.70	0.33-1.47
continuous, ^2^log	200	133	3.07	1.53-6.14	55	1.56	0.58-4.22	continuous, ^2^log	277	164	1.09	0.61-1.95	98	1.08	0.53-2.20
**IGF-II,** μg/l								**IGF-II,** μg/l							
<711	58	25	1.00		15	1.00		<843	80	36	1.00		20	1.00	
711-803	53	32	1.70	0.81-3.56	12	0.93	0.33-2.63	843 - 958	77	38	1.07	0.58-1.98	21	1.06	0.49-2.30
804-929	46	38	2.24	1.06-4.72	15	1.66	0.58-4.68	959 - 1096	58	49	1.82	0.99-3.33	26	2.11	1.00-4.47
>929	43	38	2.39	1.13-5.03	13	0.90	0.30-2.68	>1096	62	41	1.06	0.57-1.96	31	1.80	0.86-3.76
continuous, ^2^log	200	133	2.68	1.06-6.79	55	0.71	0.18-2.84	continuous, ^2^log	277	164	1.08	0.51-2.26	98	2.49	0.99-6.31

Logistic regression models were adjusted for age, BMI (<25.0, 25.0-29.9,≥30.0), physical activity during leisure time (sedentary, moderate, regular exercise), smoking (never, former, current), socioeconomic status (low, middle, high, self-employed) and blood pressure (normal blood pressure and no hypertension treatment vs high blood pressure and/or hypertension treatment).

The OR for T2D associated with low adiponectin was not affected by the adjustment for confounding risk factors in contrast to that of IGFBP-1 and -2, which decreased after extended adjustment (**Table 2** and **Supplemental Table S1**).

### Men

The control men, who stayed NGT, demonstrated compared to the control women higher concentrations of insulin and IGF-II (p<0.001) and lower levels of fasting adiponectin, IGFBP-1 and IGFBP-2 (p<0.001) (**Table 1**). The glucose levels were not different.

At baseline all subjects had normal fasting glucose, OGTT and blood pressure. Significant differences were observed in all continuous parameters except for age and serum IGF-I levels in those NGT men, who later developed prediabetes or T2D compared to controls, who stayed NGT (**Table 1**). At baseline FHD was reported in 63% of men, who later developed prediabetes, and in 77% of the men, who developed T2D. BMI and waist measurements were higher in those who later developed prediabetes compared to controls, but in contrast to women, with no further increments in those who later developed T2D. Fasting and 2h OGTT plasma glucose levels differed at baseline between men, who later developed prediabetes or T2D (**Table 1**). As for women, levels of adiponectin, IGFBP-1 and IGFBP-2 were significantly lower at baseline in those who later developed prediabetes or T2D as compared to controls.

Among men, who later developed T2D, there were more sedentary activities and more current smokers (**Table 1**). Hypertension was more common in those, who later developed prediabetes or T2D.

At follow up, 10 years later, the number with known FHD had increased to 66% of those with prediabetes and to 82% of those with T2D.

After adjustment for known risk factors, significant ORs for future T2D were obtained for the lowest quartiles of IGFBP-1 (OR:14.89), IGFBP-2 (OR:7,63) and adiponectin (OR: 2.35). Neither IGF-I nor IGF-II predicted AGT. For the prediction of prediabetes, only IGFBP-1 (OR:13.44) and IGFBP-2 (OR:4.03) yielded significant ORs (**Table 2**). Extended adjustment for confounding risk factors did not significantly alter the ORs for T2D or prediabetes (**Table 2** and **Supplemental Table S1**).

### Combining biomarkers and comparisons to BMI

In both women and men, low serum levels of adiponectin, IGFBP-1 and IGFBP-2 showed a concentration dependent risk to develop T2D (**Table 2**). When included in the same regression model and following multivariate analysis and adjustment of established risk factors, only adiponectin and IGFBP-2 remained significantly and independently associated with future prediabetes and T2D in women, while only IGFBP-1 remained associated with future prediabetes and T2D in men (**Table 3**).

**Table 3:** Odds ratios (ORs) for decreasing values of IGFBP-2, adiponectin and IGFBP-1 measured at baseline and included in the same regression model in the association to development of prediabetes and type 2 diabetes at follow-up in women and men.

	NGT	Prediabetes				Type 2 diabetes			
	n	n	OR^c^	95% CI	p	n	OR^c^	95% CI	p
WOMEN									
Model 1^a^	200	133				55			
IGFBP-2, ^2^log			3.19	1.96-5.19	<0.0001		3.03	1.50-6.13	0.0021
Adiponectin, ^2^log			2.74	1.69-4.42	<0.0001		4.90	2.29-10.51	<0.0001
IGFBP-1, ^2^log			1.47	0.99-2.16	0.0535		2.39	1.30-4.37	0.0499
Model 2^b^	200	133				55			
IGFBP-2, ^2^log			2.70	1.59-4.57	0.0002		3.04	1.23-7.52	0.0158
Adiponectin, ^2^log			2.40	1.45-3.97	0.0007		7.75	2.94-20.43	<0.0001
IGFBP-1, ^2^log			1.32	0.86-2.02	0.2070		1.39	0.69-2.79	0.3629
MEN									
Model 1^a^	277	164				98			
IGFBP-2, ^2^log			1.28	0.85-1.95	0.2392		1.36	0.83-2.25	0.2247
Adiponectin, ^2^log			1.06	0.71-1.57	0.7768		1.43	0.89-2.29	0.1423
IGFBP-1, ^2^log			3.21	2.33-4.43	<0.0001		2.86	1.97-4.15	<0.0001
Model 2^b^	277	164				98			
IGFBP-2, ^2^log			1.21	0.77-1.89	0.4139		1.60	0.93-2.77	0.0924
Adiponectin, ^2^log			0.98	0.64-1.48	0.9068		1.25	0.76-2.05	0.3901
IGFBP-1, ^2^log			3.05	2.18-4.25	<0.0001		2.62	1.73-3.96	<0.0001

^a^Model 1: adjusted for age (36-40, 41-45, 46-50, 51-56 yrs)

^b^Model 2: adjusted for age, BMI (<25.0, 25.0-29.9, ≥30.0), physical activity during leisure time (sedentary, moderate, regular exercise), smoking (never, former, current), socioeconomic status (low, middle, high, self-employed) and blood pressure (normal blood pressure and no hypertension treatment vs high blood pressure and/or hypertension treatment).

^c^OR for the association between glucose tolerance and decreasing values of the variable.

**Figure 1:**
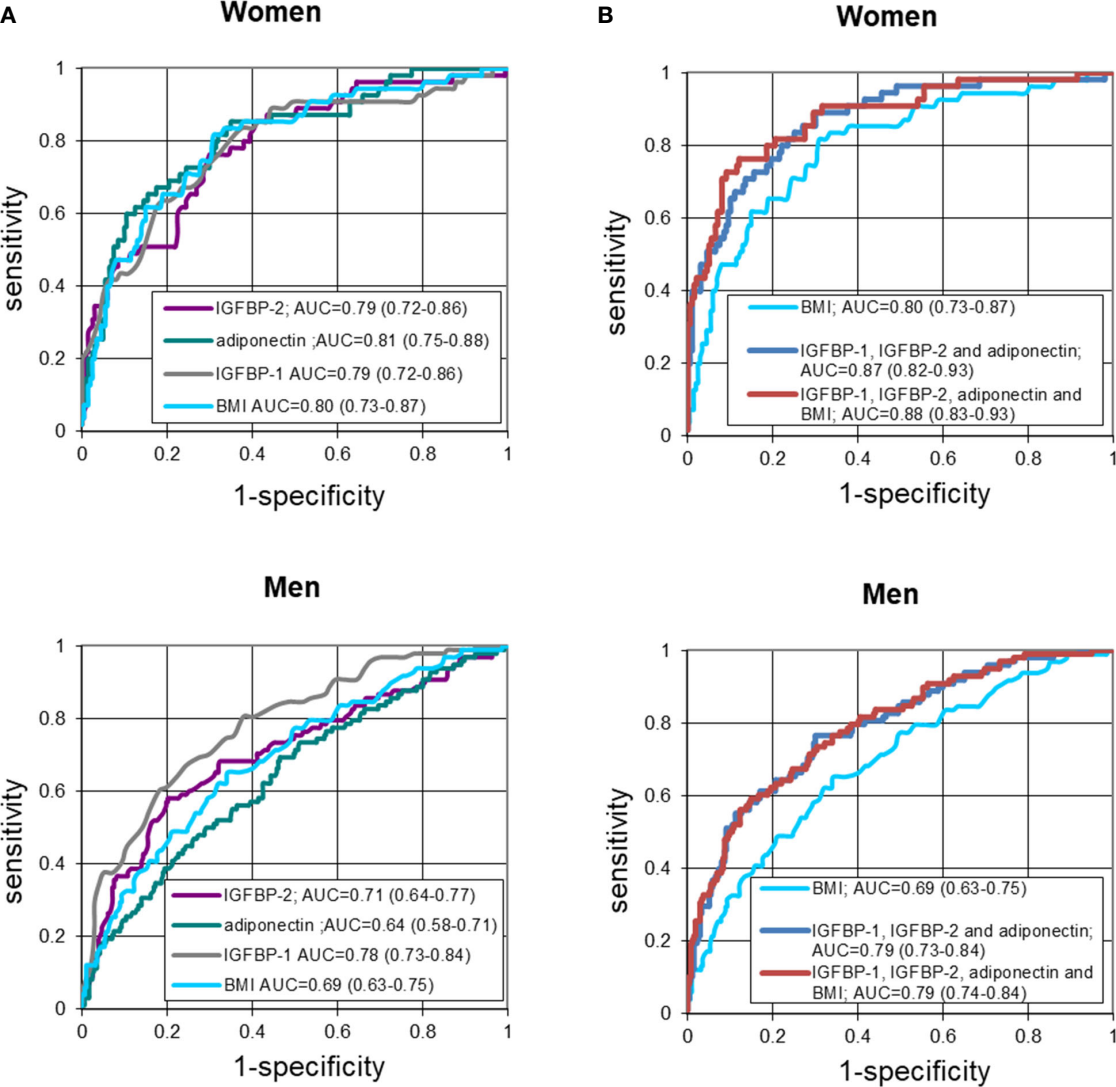
**(A)** Receiver-operating characteristic (ROC) curves of fasting IGFBP-2, adiponectin, IGFBP-1 and BMI in detecting T2D in women and men. Logistic regression models not adjusted for confounders. **(B)** Receiver-operating characteristic (ROC) curves of fasting IGFBP-2, adiponectin and IGFBP-1 combined, as compared to BMI alone or included, in detecting T2D in women and men. Logistic regression models not adjusted for confounders.

ROC curves for the three variables adiponectin, IGFBP-1 and IGFBP-2 were evaluated in comparison with the ROC curve for BMI. In women, the AUC-ROCs were; 0.81, 0.79, and 0.79 for adiponectin, IGFBP-1, and IGFBP-2, respectively, compared to 0.80 for BMI (**Figure 1A**). These AUCs did not differ significantly. In men, the corresponding AUC-ROCs were 0.64, 0.78, 0.71, respectively, and for BMI 0.69 with the value for IGFBP-1 being significantly higher compared to the other proteins (p<0.001 to p=0.003). When adiponectin, IGFBP-1, and IGFBP-2 were combined the ROC-AUC reached 0.87 for women and 0.79 for men, which was significantly higher than for BMI alone, p=0.049 for women and p=0.002 for men. Including BMI in the model did not markedly change the AUC ROCs (**Figure 1B**).

Thus, in women, the sensitivity was similar for BMI and the three biomarkers. However, when adiponectin, IGFBP-1 and IGFBP-2 were combined, they were together more sensitive than BMI. In contrast to women, in men IGFBP-1 showed a significantly higher sensitivity than adiponectin, IGFBP-2 or BMI. When combined the three biomarkers were as in women resulting in a higher sensitivity than BMI.

## Discussion

In this study investigating the risk for healthy middle-aged NGT men and women, many with a positive FHD, to develop prediabetes or overt T2D over a period of 8-10 years, we focused on biomarkers of insulin sensitivity and insulin production, i.e. adiponectin, IGFBP-1, IGFBP-2, IGF-I and IGF-II; proteins being linked to AGT (5–10, 15–18, 21, 22, 25–29). Low plasma concentrations of adiponectin, IGFBP-1 and -2 associated with future AGT in both genders. Included in the same regression model following adjusted multivariate analyses low levels of adiponectin and IGFBP-2 remained independently associated with development of AGT in women, while low levels of the liver specific IGFBP-1 remained the only associated biomarker in men. Thus, the biomarkers showing insulin resistance were present at baseline in those NGT men and women, who 10-8 years later developed AGT. Finally, in ROC curve analyses the combination of adiponectin, IGFBP-1 and IGFBP-2 yielded AUCs higher than that of BMI in both men and women, and inclusion of BMI did not increase the AUCs any further, although BMI is an accepted surrogate for HOMA-IR (WHO Expert Consultation December 2008, ISBN 9789241501491). Thus, this longitudinal study performed in a well-described large cohort confirms that in normoglycemic women, low levels of adiponectin and IGFBP-2 are predictors of T2D (18, 21, 22, 26–28), whereas in normoglycemic men, low levels of IGFBP-1 predict future T2D (16).

The finding that low adiponectin, which reflects unhealthy adiposity (24, 38), was the strongest predictor (OR:29.42) for future T2D in overweight NGT women agrees with our previous finding of waist index as a stronger predictor than both IGFBP-1 and insulin in women (17). Our finding also agrees with other studies (24–27, 39–41) showing that increasing amount of visceral adiposity associates with insulin resistance and a great risk to develop AGT in women. Noteworthy, the role of adiponectin was recently supported by genetic studies (38). Visceral adipose tissue secretes relatively high amounts of inflammatory cytokines, which inhibit the adiponectin secretion and amplify the risk for AGT (15, 25–28). In a population-based study of gender differences, adiponectin correlated inversely with markers of inflammation (CRP and sedimentation rate) in women but not in men (39). This is in line with the report by Saltevo et al. showing that inflammatory markers are higher in women than in men with AGT (42).

In NGT women the OR for prediabetes was highest for low IGFBP-2 (OR:7.48), closely followed by low adiponectin (OR:6.06). These NGT women had at baseline lower BMI, waist circumference, fasting glucose and insulin than those who later developed T2D. Low IGFBP-2 levels suggest leptin resistance and excess nutrition (19, 20). IGFBP-2 has achieved increasing interest due to its metabolic involvement (6, 40, 43, 44). Preclinical studies show that IGFBP-2 inhibits adipogenesis (19) and protects against the development of obesity and insulin resistance (6, 40, 41, 43, 44). Several studies demonstrate that IGFBP-2 is inversely related with BMI and insulin (6, 18–21, 23, 40, 43). However, the mechanisms by which IGFBP-2 exert its beneficial metabolic actions remain to be fully clarified. One possible explanation is that IGFBP-2 acts through sequestration of IGFs. However, this mode of action appears less likely. Another, and in our view more likely explanation is that IGFBP-2 possesses IGF-independently beneficial metabolic effects (6, 19) as recently reviewed (43–45). Supportive of this, approximately 50% circulating IGFBP-2 is indeed not carrying an IGF molecule (41).

In contrast to our findings in women, the strongest predictor of both prediabetes and T2D in NGT men was a low fasting IGFBP-1. This suggests that hepatic and probably whole-body insulin resistance and peripheral hyperinsulinemia (13, 14, 16, 19), are the most important factors predicting development of AGT in men. Fasting and 2h OGTT plasma glucose levels were the only variables that differed at baseline between the NGT men, who later developed prediabetes or T2D. It was not insulin, BMI or waist circumference as in women. These findings suggest that in NGT men with FHD, who later developed T2D, there was an early involvement of dysfunctional beta cells, being less able to further increase insulin secretion to compensate for increased glucose and hepatic insulin resistance indicated by low IGFBP-1 levels (14). This is most probably genetically determined. Interestingly, in the present NGT control group, fasting IGFBP-1 levels in men without FHD were higher than the fasting levels found in NGT control men with FHD (16). Thus, FHD was associated with lower fasting IGFBP-1 in NGT men, who stayed NGT. The fasting levels were further reduced in those, who later developed AGT. Genetic factors explain 35% of IGFBP-1 serum concentrations (46). Thus, the road to prediabetes and T2D appears to be different in several ways when comparing men and women.

IGFBP-1 functions both as an IGF-regulating protein, and as an IGF-independent protein with effects similar to IGFBP-2 (6, 43–45). Due to its RDG sequence, IGFBP-1 like IGFBP-2 can bind to an integrin receptor, which stimulates metabolic and anabolic pathways (6, 43–45). IGFBP-1 and IGFBP-2 have effects *via* inhibition of the IGF effect on adipose tissue, where they inhibit preadipocyte expansion and differentiation and lipid accumulation (11, 44, 47). IGFBP-2 has a direct effect *via* its heparin-binding domain-2 on adipogenesis, especially visceral. Mice overexpressing IGFBP-1 in endothelial cells were characterized by stimulated NO synthesis and protection against development of AGT *via* its RDG sequence (6, 45, 48). Protection against T2D has also been seen in transgene mice overexpressing IGFBP-2 and in humans overexpressing IGFBP-1 and adiponectin, respectively (40, 48–51). Thus, these three proteins could *via* cellular effects be protective against AGT and when decreased production this may be involved in the pathogenesis of AGT (6, 27, 28, 40, 43–45, 48–51).

After adjustment, the associations of high baseline levels of total IGF-I and IGF-II, respectively, with future AGT were weak and only present in women. IGF-II serum levels are elevated in obesity, irrespective of concomitant presence of T2D, and decline after diet-induced weight loss (4). This association with obesity can explain the present finding. Previous studies in cohorts consisting of men and women have shown inconsistency in the predictive value of serum IGF-I for incident T2D (5, 7, 8, 18). However, when only females were studied, an elevated serum IGF-I predicted gestational diabetes and T2D (18, 22, 52). IGF can stimulate adiposity if IGFBP-1 and -2 are suppressed (47), a strong risk factor for AGT in women. High serum levels of *free* IGF-I levels have indeed been shown to predict AGT, which is in line with our findings of high total IGF-I (positively correlated with free IGF-I) and low IGFBP-1 (negatively correlated with free IGF-I) as predictors of prediabetes and T2D in women (18, 53).

The present findings support that hyperinsulinemia and insulin resistance exists in healthy NGT subjects with FHD long before the appearance of prediabetes and overt T2D (54). It also suggests that in NGT women with FHD the increasing amount of unhealthy adiposity drives the development to T2D concomitant with a declining beta-cell function (17).

Our study has limitations. First, all studied subjects were Caucasians. Furthermore, due to the study design, the cohort was enriched with subjects having FHD, our findings cannot be generalized to the general population. Additionally, we did not analyze lipids, HbA1c or controlled for HOMA-IR, which could have added further value to the study. However, we have previously shown that fasting IGFBP-1 was a stronger predictor than both fasting insulin and glucose (16, 17). We controlled for BMI, a known strong risk factor, which is closely associated with HOMA-IR. Moreover, the selected biomarkers have previously been shown to associate well with HOMA-IR (e.g. 5, 7, 9, 13, 15, 18, 23, 25).

The strengths of our study were that OGTT were performed in all subjects at baseline and at follow-up to confirm glucose tolerance. The findings were controlled for well-established risk factors for T2D. Finally, our studied biomarkers may have direct pathogenic effects on the development of AGT, more than BMI and HOMA-IR *per se*.

In conclusion, among biomarkers associated with insulin sensitivity and secretion, low serum adiponectin was the strongest predictor for T2D in women with FHD, whereas low serum IGFBP-1 was the strongest predictor in men. Neither IGF-I nor IGF-II showed any strong predictive value. The strongest predictor for prediabetes was in men low IGFBP-1 and in women low IGFBP-2. In both genders, the combination of adiponectin, IGFBP-1 and IGFBP-2 yielded a stronger prediction for future T2D than BMI. Thus, this study supports the concept that in subjects with FHD, a strong risk factor for AGT is in women adiposity, especially visceral, and in men hepatic insulin resistance.

## Data availability statement

The raw data supporting the conclusions of this article will be made available by the authors, without undue reservation.

## Ethics statement

The studies involving human participants were reviewed and approved by Ethics Committee at Karolinska University Hospital. The patients/participants provided their written informed consent to participate in this study.

## Author contributions

KB performed the measurements of IGFBP-1 and IGF-I and wrote the manuscript, KB, JF and C-GÖ designed the study, supervised experimental data collection, analyzed and interpreted data and edited the manuscript, AH did the statistical calculation, interpreted data and edited the manuscript, IA reviewed/edited the manuscript. JF and AF supervised and performed the measurements of IGF-II, adiponectin and IGFBP-2 and edited the manuscript. KB is the guarantor of this work and as such had full access to all the data in the study and takes responsibility for the integrity of the data and the accuracy of the data analysis. All authors contributed to the article and approved the submitted version.

## Supplementary material

Supplemental Flow diagram Study design Baseline and follow-up study of men and women in Stockholm Diabetes Prevention Program and selection for the present study. FHD, family history of diabetes; T2D, type 2 diabetes; NGT, normal glucose tolerance. *Only subjects in whom heredity for diabetes was certain at follow-up are included. **Included are only subjects having data on all variables. ***These subjects were randomly selected among subjects having NGT and negative FHD at both baseline and follow-up. ****Subjects having only impaired fasting glucose were not included in the present study.
