# Supplementary material for: Adiponectin, IGFBP-1 and -2 are independent predictors in forecasting prediabetes and type 2 diabetes
Source: Front Endocrinol (Lausanne). 2023 Jan 5;13:1092307. doi: 10.3389/fendo.2022.1092307 (PMC9849561; doi:10.3389/fendo.2022.1092307)
Supplement: Supplemental Flow diagram — Study design Baseline and follow-up study of men and women in Stockholm Diabetes Prevention Program and selection for the present study. FHD, family history of diabetes; T2D, type 2 diabetes; NGT, normal glucose tolerance. *Only subjects in whom heredity for diabetes was certain at follow-up are included. **Included are only subjects having data on all variables. ***These subjects were randomly selected among subjects having NGT and negative FHD at both baseline and follow-up. ****Subjects having only impaired fasting glucose were not included in the present study. [file DataSheet_1.docx]

Excluded

Women: 466

**Follow-up study**

**Men 2002-04**

**Women 2004-06**

*Invited to health examination 2, including OGTT, body measures, blood pressure and questionnaires*

Total responders:

Men: 2,227 Women: 3,205

*M*

Responders:

Men: 10,236 (79%)

Women: 16,481 (85%)

FHD+

Men: 2,106

Women: 3,583

FHD-

Men: 3,329

Women: 4,296

Gestational diabetes

Women: 424

Age-adjusted sample

Men: 2,424

Women: 3,497

Baseline study group

Men: 3,128 Women: 4,821

***Follow-up period***

Excluded

Men: 34

Women: 125

Excluded

Men: 4,801 (47%)

Women: 8,178 (50%)

*Invited to health examination 1, including OGTT, body measurements, blood pressure and questionnaires*

Excluded

Men: 382 Women: 456

NGT

Men: Women:

FHD+ 1,422 FHD+ 2,397

FHD- 1,413 FHD- 2,153

Prediabetes

Men: Women:

FHD+ 148 FHD+ 140

FHD- 80 FHD- 68

T2D not invited to follow-up

Men: 65 Women: 63

*Invited to health examination 2, including OGTT, body measures, blood pressure and questionnaires*

Total responders:

Men: 156 Women: 124

Excluded from present study

NGT*

Men: Women:

FHD+ 998 FHD+ 1,643

FHD- 809 FHD- 1,220

Abnormal glucose tolerance*

Men: Women:

FHD+ 245 FHD+ 186

FHD- 110 FHD- 54

NGT***

Men: Women:

FHD+ 0 FHD+ 0

FHD- 277 FHD- 200

**Included in the present study****

T2D

Men: Women:

FHD+ 80 FHD+ 46

FHD- 18 FHD- 9

Prediabetes****

Men: Women:

FHD+ 108 FHD + 100

FHD- 56 FHD- 33

**Baseline study**

**Men 1992-94**

**Women 1996-98**

Postal questionnaire to all men and women aged 35-55 years, residing within 4 and 5 municipalities, respectively, in Stockholm

Men: 12,952 Women: 19,416
