# Supplementary material for: Adiponectin, IGFBP-1 and -2 are independent predictors in forecasting prediabetes and type 2 diabetes
Source: Front Endocrinol (Lausanne). 2023 Jan 5;13:1092307. doi: 10.3389/fendo.2022.1092307 (PMC9849561; doi:10.3389/fendo.2022.1092307)
Supplement: Supplementary file 2 [file Table_1.docx]

**Supplemental Table S1.** Odds ratios (ORs) for decreasing baseline values of IGFBP-2, adiponectin and IGFBP-1 and increasing baseline values of IGF-I and IGF-II in subjects having normal glucose tolerance (NGT) at baseline in the association to development of prediabetes and type 2 diabetes at follow-up compared to remaining NGT. Logistic regression models were adjusted for age.

| **WOMEN** |  |  | | |  | | |  | **MEN** |  |  | | |  | | |
| --- | --- | --- | --- | --- | --- | --- | --- | --- | --- | --- | --- | --- | --- | --- | --- | --- |
|  | **NGT** | **Prediabetes** | | | **Type 2 diabetes** | | |  |  | **NGT** | **Prediabetes** | | | **Type 2 diabetes** | | |
|  | n | n | OR | 95% CI | n | OR | 95% CI |  |  | n | n | OR | 95% CI | n | OR | 95% CI |
|  |  |  |  |  |  |  |  |  |  |  |  |  |  |  |  |  |
| **IGFBP-2,** μg/l |  |  |  |  |  |  |  |  | **IGFBP-2,** μg/l |  |  |  |  |  |  |  |
| >268 | 79 | 14 | 1.00 |  | 5 | 1.00 |  |  | >224 | 92 | 28 | 1.00 |  | 14 | 1.00 |  |
| 193-268 | 52 | 34 | 3.75 | 1.83-7.71 | 8 | 2.44 | 0.75-7.91 |  | 161-224 | 95 | 27 | 0.97 | 0.53-1.77 | 17 | 1.21 | 0.56-2.61 |
| 141-192 | 47 | 32 | 3.88 | 1.87-8.02 | 16 | 5.37 | 1.84-15.67 |  | 110-160 | 58 | 48 | 2.88 | 1.62-5.13 | 29 | 3.36 | 1.63-6.92 |
| <141 | 22 | 53 | 14.56 | 6.76-31.39 | 26 | 18.89 | 6.45-55.37 |  | <110 | 32 | 61 | 6.85 | 3.69-12.71 | 38 | 8.51 | 4.02-18.02 |
| continuous, ^2^log | 200 | 133 | 5.01 | 3.26-7.70 | 55 | 6.62 | 3.62-12.13 |  | continuous, ^2^log | 277 | 164 | 3.21 | 2.30-4.47 | 98 | 3.42 | 2.31-5.06 |
| **Adiponectin**, mg/l |  |  |  |  |  |  |  |  | **Adiponectin**, mg/l |  |  |  |  |  |  |  |
| >15.62 | 74 | 18 | 1.00 |  | 5 | 1.00 |  |  | >10.55 | 84 | 36 | 1.00 |  | 17 | 1.00 |  |
| 11.60-15.62 | 62 | 28 | 1.94 | 0.98-3.87 | 5 | 1.25 | 0.34-4.56 |  | 7.95 - 10.55 | 70 | 41 | 1.37 | 0.78-2.38 | 20 | 1.45 | 0.70-2.99 |
| 8.56-11.59 | 43 | 42 | 4.14 | 2.10-8.15 | 14 | 4.95 | 1.64-14.95 |  | 6.04 – 7.94 | 74 | 36 | 1.14 | 0.65-1.99 | 27 | 1.84 | 0.93-3.66 |
| <8.56 | 21 | 45 | 9.57 | 4.54-20.20 | 31 | 24.07 | 8.09-71.62 |  | <6.04 | 49 | 51 | 2.44 | 1.40-4.26 | 34 | 3.51 | 1.77-6.95 |
| continuous, ^2^log | 200 | 133 | 4.41 | 2.80-6.94 | 55 | 10.33 | 5.08-21.02 |  | continuous, ^2^log | 277 | 164 | 1.78 | 1.27-2.50 | 98 | 2.45 | 1.62-3.72 |
| **IGFBP-1,** μg/l |  |  |  |  |  |  |  |  | **IGFBP-1,** μg/l |  |  |  |  |  |  |  |
| >49 | 73 | 20 | 1.00 |  | 5 | 1.00 |  |  | >35 | 106 | 18 | 1.00 |  | 9 | 1.00 |  |
| 35-49 | 63 | 28 | 1.68 | 0.86-3.28 | 8 | 1.93 | 0.60-6.22 |  | 23 - 35 | 88 | 23 | 1.58 | 0.80-3.12 | 20 | 2.72 | 1.18-6.29 |
| 24-34 | 42 | 39 | 3.62 | 1.86-7.06 | 18 | 6.51 | 2.24-18.94 |  | 14 - 22 | 57 | 47 | 5.01 | 2.65-9.47 | 29 | 6.08 | 2.69-13.76 |
| <24 | 22 | 46 | 8.48 | 4.11-17.52 | 24 | 16.41 | 5.57-48.33 |  | <14 | 26 | 76 | 18.49 | 9.35-36.54 | 40 | 18.54 | 7.97-43.11 |
| continuous, ^2^log | 200 | 133 | 2.91 | 2.06-4.11 | 55 | 5.40 | 3.17-9.21 |  | continuous, ^2^log | 277 | 164 | 3.60 | 2.74-4.73 | 98 | 3.44 | 2.51-4.72 |
| **IGF-I,** μg/l |  |  |  |  |  |  |  |  | **IGF-I,** μg/l |  |  |  |  |  |  |  |
| <150 | 52 | 28 | 1.00 |  | 18 | 1.00 |  |  | <161 | 63 | 42 | 1.00 |  | 32 | 1.00 |  |
| 150-182 | 57 | 29 | 0.96 | 0.51-1.83 | 12 | 0.61 | 0.27-1.40 |  | 161 - 185 | 82 | 36 | 0.66 | 0.38-1.15 | 17 | 0.41 | 0.21-0.81 |
| 183-214 | 53 | 32 | 1.18 | 0.62-2.24 | 12 | 0.67 | 0.29-1.54 |  | 186 - 218 | 62 | 42 | 1.03 | 0.59-1.80 | 22 | 0.70 | 0.36-1.37 |
| >214 | 38 | 44 | 2.35 | 1.23-4.51 | 13 | 1.05 | 0.45-2.48 |  | >218 | 70 | 44 | 0.97 | 0.55-1.69 | 27 | 0.76 | 0.40-1.45 |
| continuous, ^2^log | 200 | 133 | 1.94 | 1.07-3.53 | 55 | 0.71 | 0.33-1.53 |  | continuous, ^2^log | 277 | 164 | 1.13 | 0.66-1.94 | 98 | 1.04 | 0.55-1.97 |
| **IGF-II,** μg/l |  |  |  |  |  |  |  |  | **IGF-II,** μg/l |  |  |  |  |  |  |  |
| <711 | 58 | 25 | 1.00 |  | 15 | 1.00 |  |  | <843 | 80 | 36 | 1.00 |  | 20 | 1.00 |  |
| 711-803 | 53 | 32 | 1.43 | 0.74-2.75 | 12 | 0.86 | 0.37-2.03 |  | 843 - 958 | 77 | 38 | 1.10 | 0.63-1.91 | 21 | 1.09 | 0.55-2.18 |
| 804-929 | 46 | 38 | 2.00 | 1.04-3.85 | 15 | 1.32 | 0.57-3.03 |  | 959 - 1096 | 58 | 49 | 1.89 | 1.10-3.37 | 26 | 1.79 | 0.92-3.52 |
| >929 | 43 | 38 | 2.18 | 1.13-4.19 | 13 | 1.23 | 0.52-2.90 |  | >1096 | 62 | 41 | 1.47 | 0.84-2.56 | 31 | 2.01 | 1.04-3.86 |
| continuous, ^2^log | 200 | 133 | 2.44 | 1.06-5.61 | 55 | 0.96 | 0.32-2.94 |  | continuous, ^2^log | 277 | 164 | 1.45 | 0.73-2.87 | 98 | 2.90 | 1.25-6.72 |
|  |  |  |  |  |  |  |  |  |  |  |  |  |  |  |  |  |

**Legend**

.

**Supplemental Table S1.** Odds ratios (ORs) for decreasing baseline values of IGFBP-2, adiponectin and IGFBP-1 and increasing baseline values of IGF-I and IGF-II in subjects having normal glucose tolerance (NGT) at baseline in the association to development of prediabetes and type 2 diabetes at follow-up compared to remaining NGT. Logistic regression models were adjusted for age.
